# Are pollinating hawk moths declining in the Northeastern United States? An analysis of collection records

**DOI:** 10.1371/journal.pone.0185683

**Published:** 2017-10-05

**Authors:** Bruce E. Young, Stephanie Auer, Margaret Ormes, Giovanni Rapacciuolo, Dale Schweitzer, Nicole Sears

**Affiliations:** 1 NatureServe, Arlington, Virginia, United States of America; 2 Department of Ecology and Evolution, Stony Brook University, Stony Brook, New York, United States of America; 3 Port Norris, New Jersey, United States of America; Northwest A&F University, CHINA

## Abstract

Increasing attention to pollinators and their role in providing ecosystem services has revealed a paucity of studies on long-term population trends of most insect pollinators in many parts of the world. Because targeted monitoring programs are resource intensive and unlikely to be performed on most insect pollinators, we took advantage of existing collection records to examine long-term trends in northeastern United States populations of 26 species of hawk moths (family Sphingidae) that are presumed to be pollinators. We compiled over 6,600 records from nine museum and 14 private collections that spanned a 112-year period, and used logistic generalized linear mixed models (GLMMs) to examine long-term population trends. We controlled for uneven sampling effort by adding a covariate for list length, the number of species recorded during each sampling event. We found that of the 22 species for which there was sufficient data to assess population trends, eight species declined and four species increased in detection probability (the probability of a species being recorded during each year while accounting for effort, climate, and spatial effects in the GLMMs). Of the four species with too few records to statistically assess, two have disappeared from parts of their ranges. None of the four species with diurnal adults showed a trend in detection probability. Two species that are pests of solanaceous crops declined, consistent with a seven-fold drop in the area planted in tobacco and tomato crops. We found some evidence linking susceptibility to parasitoidism by the introduced fly *Compsilura concinnata* (Tachinidae) to declines. Moths with larvae that feed on vines and trees, where available evidence indicates that the fly is most likely to attack, had a greater propensity to decline than species that use herbs and shrubs as larval host plants. Species that develop in the spring, before *Compsilura* populations have increased, did not decline. However, restricting the analysis to hawk moth records from areas outside of a “refuge” area where *Compsilura* does not occur did not significantly increase the intensity of the declines as would be predicted if *Compsilura* was the primary cause of declines. Forests have recovered over the study period across most of the northeastern U.S., but this does not appear to have been a major factor because host plants of several of the declining species have increased in abundance with forest expansion and maturation. Climate variables used in the GLMMs were not consistently related to moth detection probability. Hawk moth declines may have ecological effects on both the plants pollinated by these species and vertebrate predators of the moths.

## Introduction

Recent reports of declines in both managed and native pollinators have raised concern about pollination limitation in crops and natural ecosystems [1], [2], [3]. Among native pollinators, declines have been reported in bumble bees (genus *Bombus*) [4], [5], other bees [6], avian and bat pollinators, and butterflies [3]. The available data indicate that many wild pollinators have declined in occurrence and diversity (and abundance for certain species) at local and regional scales in northwestern Europe and North America [6], [3]. Concern over the plight of pollinators in the United States led to the development of a national strategy to promote the health of pollinators [7]. However, population trends in most pollinator species remain unknown. Many species are small-bodied and hard to identify in the field, are not commonly sought after by citizen naturalists, or occur in countries with limited resources for monitoring. Identifying strategies for estimating population trends in these species is challenging, but necessary to broaden our knowledge about conservation status of native pollinators.

Among vertebrate and invertebrate pollinators, most moths are easy to overlook due to their nocturnal activity. Nevertheless, moth pollinator guilds can be diverse and form the basis of complicated pollen-transfer networks [8], [9], [10]. Some plants, such as the western prairie fringed orchid (*Platanthera praeclara*), yucca (*Yucca* and *Hesperoyucca*) and senita cacti (*Lophocereus schottii*), depend exclusively on one or a small number of moth species for pollination [11], [12] [13]. Declines in moth diversity and abundance could therefore lead to disruptions in the plant communities they pollinate [14], [12].

The hawk moths (family Sphingidae), a group of relatively large-bodied and strong flying Lepidoptera, include many pollinating species that typically feed nocturnally (although some do so diurnally) at pale-colored flowers with long corollas and a sweet odor [15], [16], [17]. One species, *Manduca sexta*, is well known as a model organism for laboratory studies of animal behavior and neurobiology [18], [19]. Evolutionary biologists and ecologists recognize this group as being diverse and important in the study of insect-plant interactions in both tropical and temperate ecosystems [20], [21], [17], especially because hawk moth tongue length seems to coevolve with plant corolla length [22], [23]. Although the relationship between plants and pollinating animals is more complex than simple ‘syndromes’ [24], hawk moths are clearly able to use their typically long tongues to pollinate flowers with long corollas that exclude other potential pollinators [25]. Some plants pollinated by hawk moths are rare, including members of the orchid, lily, and evening-primrose families (Orchidaceae, Liliaceae, and Onagraceae, respectively; [26], [27], [28]), highlighting the potential conservation ramifications of hawk moth population declines.

Preliminary evidence suggests that several hawk moth species, along with members of another family of large moths, the Saturniidae, have undergone long-term declines in the northeastern U.S. and adjacent Canada [29], [30], [31], [32], [33], [34]. As for virtually all of the world’s more than one million species of insects, quantitative monitoring data are not available to document most of these declines [35]. Possible causes of declines in hawk moths include climate change, which might cause a mismatch between emergence of moth larvae and host plant leaf out; loss of habitat including host plants; forest succession, which has led to long-term compositional changes as northeastern U.S. forests recover from 19^th^ century clearing for agriculture [36], [37]; increasing levels of artificial lights at night [14]; an introduced parasitoid fly (*Compsilura concinnata*; Tachinidae) [38]; and changing agricultural practices and land use that have caused declines in hosts available for hawk moths with larvae that feed on crop plants [33]. Considering the importance of hawk moths in their ecosystems and the diversity of threats they face, the absence of monitoring data is concerning because declines could be widespread without the conservation community having a means to detect them.

*Compsilura* has been implicated in declines of numerous moths [39]. The parasitoid, native to Europe, was repeatedly introduced from 1906–1986 to control non-native gypsy moths (*Lymantria dispar*) and other pests. The multivoltine generalist parasitoid never successfully controlled gypsy moth populations, but is now known to attack 200 North American lepidopteran species, including numerous hawk moths [40], [39] [33]. Experimental evidence indicates that *Compsilura* is more effective at attacking hosts during the summer than in the spring, and is most frequently recorded attacking hosts on leaves of trees that are situated in a group or forest setting rather than shrubs or herbs [41]. The fly has been found everywhere it has been looked for in the northeastern U.S., except coastal sand dune habitats in Cape Cod and Martha’s Vineyard and Nantucket islands, Massachusetts, U.S., where sparse vegetation apparently does not support a succession of potential hosts needed by the multivoltine fly during the spring-to-fall period of activity [42].

Because of limited resources available for biological surveillance monitoring [43] targeted studies on the diverse hawk moth fauna that are unlikely to occur over broad geographical areas. In this situation, a potential substitute is an analysis of museum and private collecting records [44], [45]. Although museum and private collecting efforts will rarely have the temporal or spatial regularity of a targeted study, they have the advantage of covering century-long time scales, large geographic areas, and large numbers of species. However, estimating trends from museum records necessitates addressing issues of recording bias, since these records were often collected in an opportunistic manner largely contingent on the context of each recording event [46], [47]. One effective approach to statistically correct for uneven recorder effort in opportunistic data is to use the number of species recorded during each sampling visit (the list length, L; *sensu* [48] as a proxy for recorder effort (e.g., [49], [50], [51], [52], [53]). Adding a covariate for list length within models of temporal estimates may enable disentangling true absences from failures to detect and record given species, because list length is positively associated with recorder effort and therefore probability of detection [54], [55]. Northeastern U.S. Lepidoptera are particularly amenable to this statistical approach as they have been relatively well studied, and private collection data can supplement material in museums [33]. Furthermore, an advantage of hawk moths over many other insect groups is that most species (except for the *Sphinx gordius*-*S*. *poecila* complex) are readily identified and therefore determinations of museum specimens are reliable.

In this study, we focus on the species of hawk moths that breed in the northeastern U.S. and feed as adults and therefore putatively serve as pollinators (but see [13]). We ask whether we can detect long-term declines for any of these species, while statistically correcting for recorder effort, from museum and private collecting records. We then test five predictions of hypotheses that may explain the trends. First, we ask whether the spatial and temporal abundance of records is related to changes in climate that are predicted to be important to moths, such as cold winters, or hot or wet summers [33]. Second, we test the prediction that if *Compsilura* is an important cause of declines, then eliminating records from where the fly does not occur (e.g., the Massachusetts sand dune habitats [42]), and thus where moth declines may not have taken place, should result in more significant declines. Third, we test the prediction that species with larvae most exposed to *Compsilura*, those that feed on trees or vines, should have declined more than species with less exposed larvae feeding on understory shrubs and herbs [41]. Fourth, we tested the prediction that species that are present as larvae in May and June, before *Compsilura* populations increase seasonally, should have declined less than species active during the summer months [41], [32]. Fifth, to address whether changes in agricultural practices has led to a decline in host plant availability, we examined long-term records in the acreage of the solanaceous crops tobacco and tomato (host plants of two *Manduca* species) planted on farms in six northeastern U.S. states. The results provide the most comprehensive analysis to date of long-term population changes in a regional hawk moth fauna.

## Materials and methods

### Study area, species selection, and record compilation

**Fig 1:**
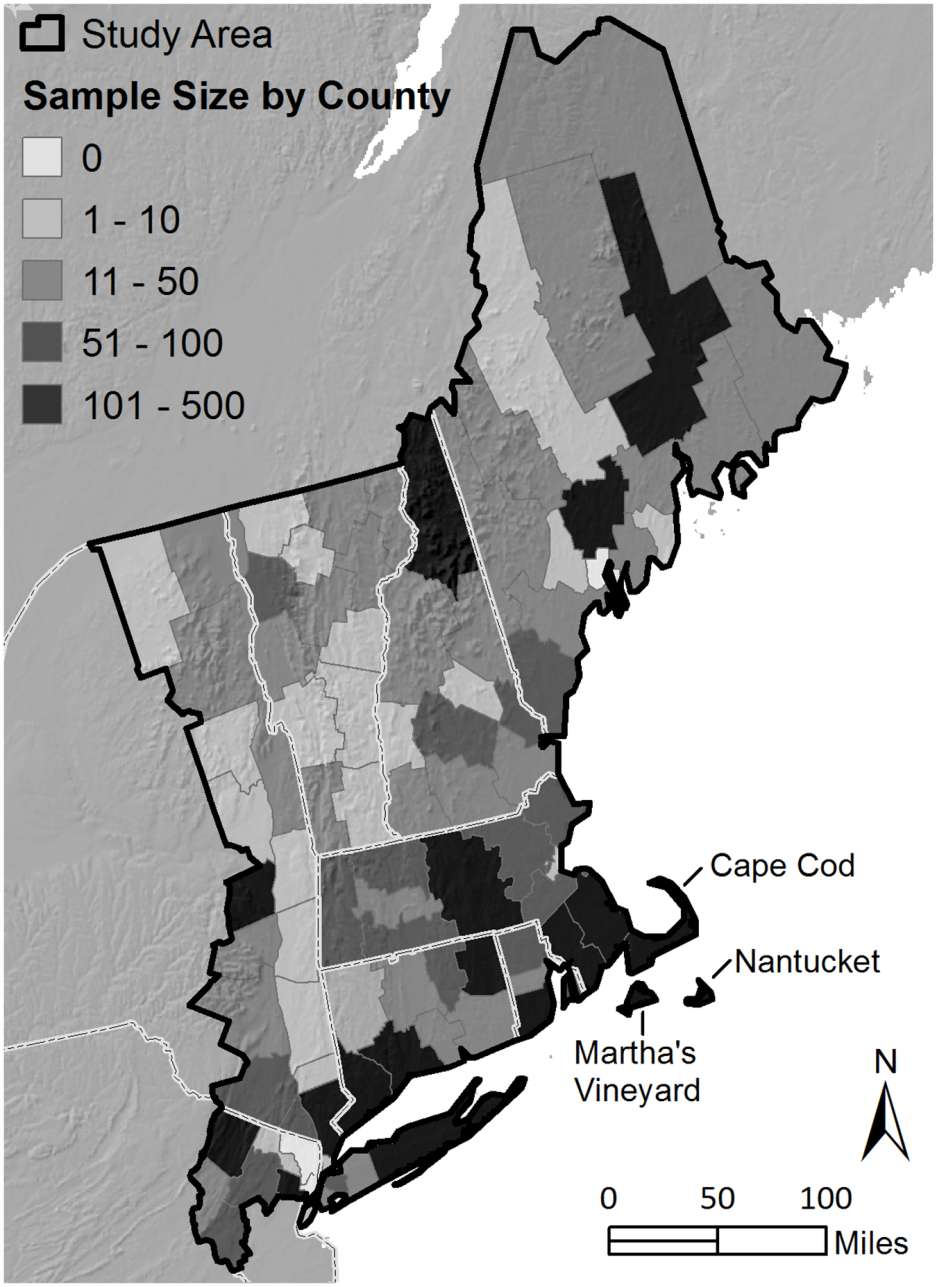
Map of study area showing the number of hawk moth records compiled from each county.

Our study area in the northeastern U.S. included the six New England states (Maine, New Hampshire, Vermont, Massachusetts, Rhode Island and Connecticut) plus nearby New York (Long Island and the counties bordering the Hudson River) and northern New Jersey (between 40° 7’ N and 47° 26’ N and 66° 58’ and 74° 56’ W; Fig 1). Within this area, we defined records from all but the outer tip of Cape Cod, Nantucket, and Martha’s Vineyard, Massachusetts, as “Exposed to *Compsilura*” areas (where moths are potentially subject to *Compsilura* attack) and the remaining records as “Refuge from *Compsilura*” areas according to [42]. We focused on the 26 species of hawk moths that breed in this area and feed as adults and therefore are presumed to pollinate plants ([25]; Table 1). Additional species occur in the study area as migrants or are nonfeeding as adults. Due to the difficulty in distinguishing *Sphinx gordius* from *S*. *poecila*, we grouped records for these two taxa and consider them as a single species for the purpose of species tallies. The two species have similar sizes, life histories and host plants [25]. We also compiled information relevant to the hypotheses we tested, including flight activity (nocturnal, which included crepuscular, or diurnal), seasonality of flight period (as an indication of the timing of the preceding larval development period when individuals are subject to parasitoidism) and the growth form of larval food plants from [25] (Table 1).

**Table 1:** Species of hawk moths, their size and natural history, and the sample sizes of records compiled for analysis of species declines in the northeastern United States.

Species^a^ and Forewing Length (mm)	Activity^b^	Host Plant Habit (Hosts)^c^	Flight Period^d^	Records^e^	Lists^f^
**Macroglossinae**					
*Amphion floridensis* (22–24)	Diurnal	Vines (Vitaceae)	May-early July	217	27
*Darapsa choerilus* (27–34)	Nocturnal	Shrubs (Ericaceae, Caprifoliaceae)	June-August	817	367
*Darapsa myron* (26–32)	Nocturnal	Vines (Vitaceae)	June-July	454	155
*Darapsa versicolor* (32–38)	Nocturnal	Shrubs (Hydrangeaceae: *Hydrangea*, Lythraceae: *Decodon*, Rubiaceae: *Cephalanthus*)	June-July	105	29
*Deidamia inscriptum* (22–25)	Nocturnal	Vines (Vitaceae)	May-June	506	191
*Eumorpha achemon* (42–49)	Nocturnal	Vines (Vitaceae)	July-September	69	30
*Eumorpha pandorus* (47–52)	Nocturnal	Vines (Vitaceae)	July-August	242	107
*Hemaris diffinis* (16–22)	Diurnal	Shrubs (Caprifoliaceae: *Lonicera*)	May-August	253	44
*Hemaris gracilis* (17–20)	Diurnal	Shrubs (Ericaceae: *Vaccinium*)	May-early July	53	12
*Hemaris thysbe* (23–28)	Diurnal	Shrubs (Caprifoliaceae: *Viburnum*, *Lonicera*)	May-August	410	52
*Hyles gallii* (25–43)	Nocturnal	Herbs (Onagraceae: *Chamerion*, Rubiaceae: *Galium*)	June-July	166	26
*Hyles lineata* (29–47)	Nocturnal	Herbs (Onagraceae: *Oenothera*, Portulacaceae: *Portulaca*)	July-Sept	137	32
*Proserpinus flavofasciata* (16–19)	Diurnal	Herbs (Onagraceae: *Chamerion*)	May-June	2	1
*Sphecodina abbottii* (27–31)	Nocturnal	Vines (Vitaceae)	May-June	356	132
**Sphinginae**					
*Dolba hyloeus* (22–30)	Nocturnal	Shrubs (Aquifoliaceae: *Ilex*)	June-August	286	118
*Lintneria eremitus* (27–37)	Nocturnal	Herbs (Lamiaceae)	July-August	120	36
*Manduca jasminearum* (40–50)	Nocturnal	Trees (Oleaceae: *Fraxinus*)	July-early August	66	8
*Manduca quinquemaculatus* (52–57)	Nocturnal	Herbs (Solanaceae; mostly crops)	June-September	121	24
*Manduca sexta* (51–56)	Nocturnal	Herbs (Solanaceae; mostly crops)	July-August	163	57
*Sphinx canadensis* (33–43)	Nocturnal	Trees (Oleaceae: *Fraxinus*)	June-August	44	4
*Sphinx chersis* (38–55)	Nocturnal	Trees (Oleaceae: *Fraxinus*)	June-August	204	36
*Sphinx drupiferarum* (45–52)	Nocturnal	Trees (Rosaceae: *Amelanchier*, *Malus*, *Prunus*)	May-July	166	50
*Sphinx gordius/S*. *poecila* (32–43)	Nocturnal	Shrubs (Ericaceae, Myricaceae, Rosaceae: *Spirea*)	May-July	1253	402
*Sphinx kalmiae* (42–48)	Nocturnal	Trees (Oleaceae: *Fraxinus*, *Syringa*)	June-July	364	94
*Sphinx luscitiosa* (26–37)	Diurnal	Trees and shrubs (Betulaceae: *Betula*, Salicaceae: *Populus*, *Salix*)	May-June	40	2

^a^ Species are listed alphabetically within subfamilies.

^b^ Species with crepuscular or a combination of diurnal and nocturnal activity were classed as nocturnal.

^c^ Principal hosts listed by [25] that occur within the study area; some species may use additional hosts.

^d^ Populations in the northern portion of the study area are active somewhat later in the season.

^e^ Number of individuals in collections examined with collection year and county data.

^f^ Unique combinations of locality, date, and collector name that included the species. Species with fewer than ten lists were excluded from the analyses.

We compiled information on species, date collected, locality, county, and collector for our focal species from specimens held at most northeastern U.S. natural history collections known to have significant holdings of hawk moths. We also canvassed the community of academic and private hawk moth collectors to obtain additional records. The Acknowledgments provides a full list of the nine museum and 14 private collections utilized. Due to a preponderance of incomplete data on labels of specimens collected prior to 1900 and incomplete processing and databasing of more recent collections, we restricted the sample to include records from 1900 through 2012. All records are publicly available at http://www.natureserve.org/conservation-tools/locality-data-northeastern-us-hawk-moths and at Figshare (https://doi.org/10.6084/m9.figshare.5435950.v1).

### Climate data

To understand possible relationships between climate and moth population trends, we associated climate values with corresponding moth records by county and year. For each year in the study period (1900–2012), we derived three bioclimatic variables (named following the terminology of the WorldClim dataset, www.worldclim.org: Bio6, minimum temperature of coldest month; Bio10, mean temperature of the warmest quarter; and Bio18, precipitation during the warmest quarter) from monthly temperature and precipitation following the method of [56]. These variables represent extreme conditions that can influence overwintering survival of larvae (Bio6), and survival of adults (Bio10 and Bio18). Directional changes in these variables, such as could be caused by global warming, could result in changes in abundance of moths in the northeastern U.S. portion of their ranges. The source data consist of 800-meter resolution gridded surfaces from the PRISM LT71 dataset [57], [58]. We calculated the mean of each bioclimatic variable for each county in the study area for each year of the study period, and then associated these values with corresponding moth records by county and year for analysis.

### Extent of tobacco and tomato farming

To explore the possible relationship between the availability of the solanaceous crops tobacco and tomato as host plants and population trends in the species *Manduca sexta* and *M*. *quinquemaculatus*, we summarized records from the U.S. Department of Agriculture (USDA) on trends in the area planted in these crops on farms over the course of the study period [59], [60]. Because crop statistics are reported by state, we included only the six New England states, which are entirely included in the study area, in the compilation.

### Analyses and assumptions

We first aggregated species records into species “lists” with unique combinations of locality, date, and collector name. We assumed species lists represented a finite recording event from which we could derive estimates of recording effort, and that all species detected were collected and deposited in a collection. For each species list, we estimated recording effort using *1)* the total number of individuals collected, regardless of species, and *2)* the total number of species recorded within a list (or list length). We assumed that the number of records and species collected was related to recording effort, such that a higher number of records and species collected indicate a higher recording effort [61], [62]. We excluded lists with fewer than two recorded species, as these lists were particularly likely to represent incomplete samples [61]. The final datasets we analyzed consisted of 80 and 750 species lists, for diurnal and nocturnal species respectively. To ensure convergence during model calibration, we focused on the four diurnal and 17 nocturnal species recorded in at least ten species lists. We qualitatively discuss the records available for the remaining two diurnal and two nocturnal species.

We modeled whether each species was recorded or not in each list—the species’ reporting rate *y* (*sensu* [63])–as a function of the probability of detection *p* using logistic generalized linear mixed models (GLMMs). The probability of detection *p* estimates the likelihood that a species was reported on a list during a recording event, given that the species was actually present in that county in that year. Our primary goal was to estimate how the reporting rate of each species varied across years—a measure of the temporal trend in species’ occurrence. Trends in reporting rate across years may result not only from ecological processes but also from changes in recorder effort. Therefore, we accounted for variation in recorder effort among lists by including a list length term—the log of the number of species recorded in a list (*L*)–as well as a term for the total number of records collected in a list (*records*). The inclusion of a year term in conjunction with list-based proxies for recorder effort has previously been reliably used to estimate species’ trends from opportunistic data such as museum records (e.g. [49], [50], [51], [52], [53]). To examine the potential effects of climate on temporal trends, we also included a linear term for each of the bioclimatic variables examined. We checked for multicollinearity among continuous predictor variables using variance inflation factors and pairwise Spearman’s rank correlations. All variance inflation factors were lower than 2.1 and all pairwise rank correlations were lower than 0.62 (S1 Table); taken together, both measures indicate low multicollinearity among continuous predictor variables. Finally, we controlled for unaccounted spatial relationships among sites by including a random effect of county, thereby estimating a different intercept for each county *i*. We standardized all continuous predictors—by subtracting the mean and dividing by the standard deviation—to facilitate the interpretation of the relative importance of model coefficients [64].

For each species, we thus considered the following model of reporting rate *y* at site *s* in year *t* for list *l* as a function of the probability of detection *p* (modified from [63]):

$$\begin{array}{l} y_{stl}\;\sim \;Bernouilli\left(p_{st}\right)\\ logit\left(p_{st}\right)=\;\alpha +\;\beta_{1}\times year+\beta_{2}\times \text{log}\left(L_{stl}\right)+\beta_{3}\times records_{stl}+\;\beta_{4}\times bio6_{st}\;+\beta_{5}\times bio10_{st}+\beta_{6}\times bio18_{st}+county_{i} \end{array}$$ (1)

where the parameter *β*_*1*_ estimates the yearly change in probability of detection across lists; *β*_*2*_ estimates how probability of detection varies with the length of each list; *β*_*3*_ estimates how probability of detection varies with the number of records collected; *β*_*4*_, *β*_*5*_ and *β*_*6*_ relate to the three bioclimatic variables; and *county* is a random effect of mean zero, where variance is estimated across counties.

We used multi-model inference to identify the set of candidate models best explaining reporting rate for each species and ranked them based on their relative weight of evidence [65], [66]. All candidate models included the variables aimed at controlling for recorder bias—the fixed terms *L* and *records* and the *county* random term—but varied in their inclusion of the single and combined fixed effects of *year*, *bio6*, *bio10*, *and bio18*. We ranked models using the Akaike Information Criterion correction for small sample sizes (AICc: [65]). For each candidate model, we quantified the probability that it was the best model given the data using AICc weights (AIC_w_). For species where no single model was overwhelmingly supported (i.e., no model with AIC_w_ > = 0.9), we considered the model set comprising the models with Akaike weights of at least 5% of the best model weight (an evidence ratio of 0.05; [67]). We used this set to calculate model-averaged coefficients and standard errors for each predictor appearing at least once in the set; we averaged coefficients only over the models in which each predictor appeared. For a summary of the models selected for each species see S2 and S3 Tables. All multi-model inference analyses were run using the MuMIn package in R (functions *pdredge* and *model*.*avg*; [68], [69]).

To analyze the relationship between host plant habit (trees and vines versus herbs and shrubs) and whether species declined, we used a two-tailed Fisher’s Exact Test.

## Results

**Fig 2:**
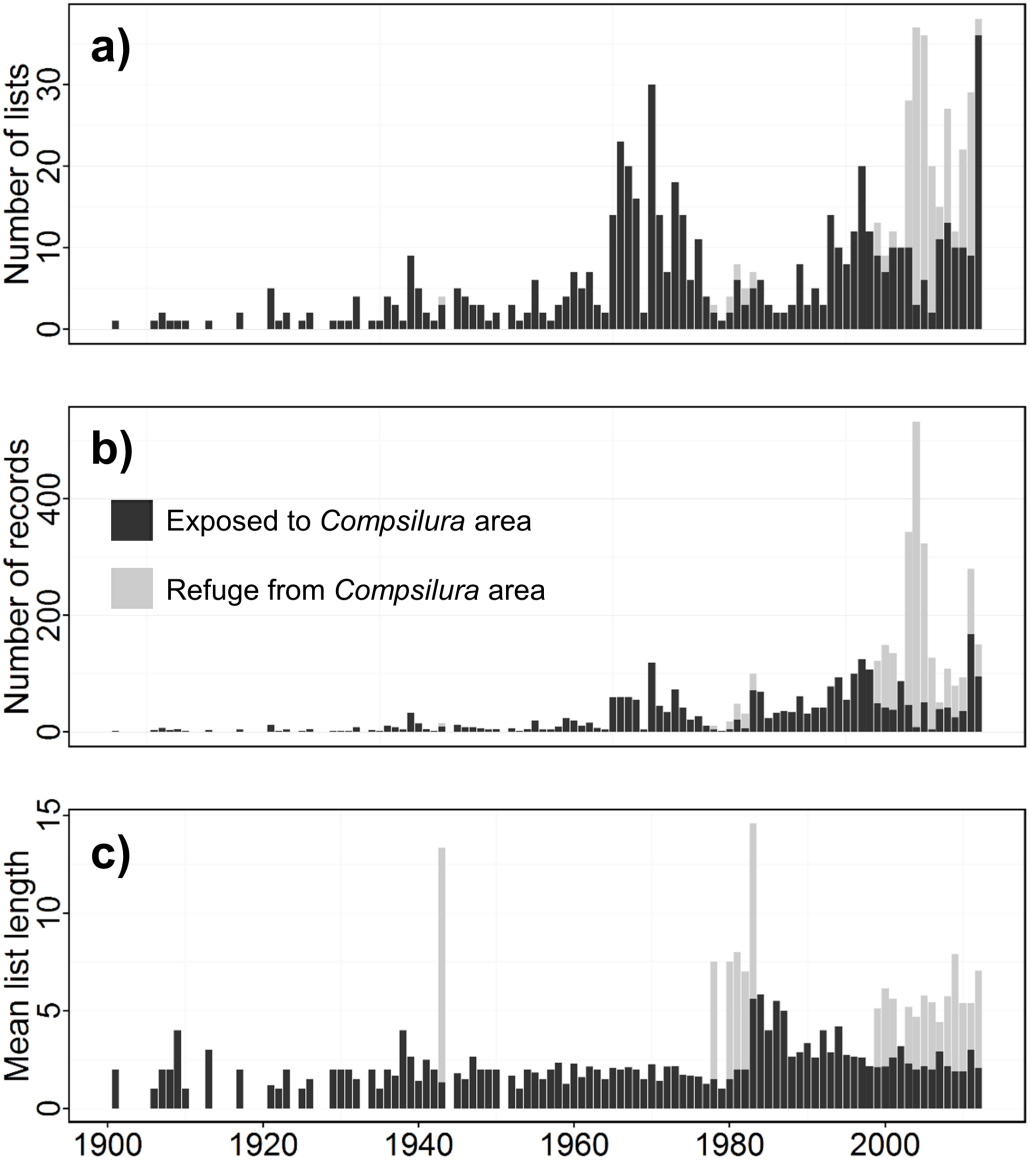
Sample sizes of lists of northeastern U.S. hawk moth records, number of lists, and mean list length, or the number of species recorded in each list, for the period 1900–2012. Records from Refuge from *Compsilura* areas, indicated in gray, are from the outer tip of Cape Cod, Nantucket, and Martha’s Vineyard where the *Compsilura concinnata* parasitoid is presumed not to occur due to unsuitable habitat [42]; Exposed to *Compsilura* areas, where caterpillars are subject to *Compsilura* attack, are everywhere else.

We compiled a total of 6,614 records of the focal species within the study area, comprising 2,787 lists of unique combinations of locality, date, and collector. Numbers of observations and lists were smaller prior to the mid-1960s than afterwards, whereas average list length ranged mostly from 1–2 species until an increase in the late 1970s (Fig 2).

**Fig 3:**
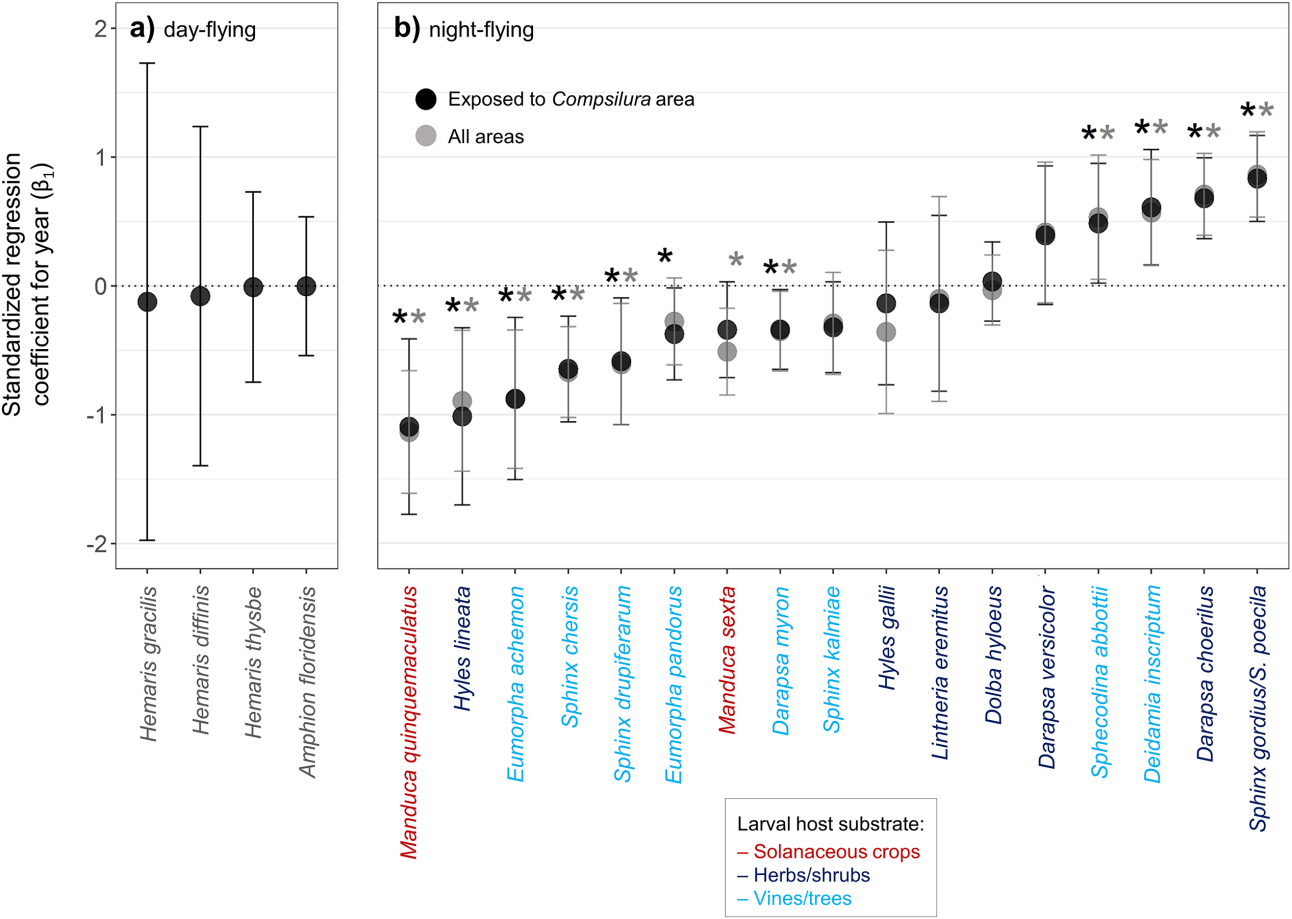
Trend in relative detection probability 1900–2012 in the northeastern U.S. for (a) diurnal and (b) nocturnal hawk moth species. Detection probability refers to the probability of a species being recorded during each year while accounting for effort, climate, and spatial effects using logistic generalized linear mixed models. Closed circles represent means of the standardized regression slope for year and error bars represent 95% confidence intervals. Declining species are those with error bars completely below zero, increasing species are those with error bars completely above zero, and species with no trend are those with error bars that overlap zero. For nocturnal species, dark gray and light gray asterisks represent significant (i.e., error bars do not overlap zero) temporal trends based on Exposed to *Compsilura* and Exposed to *Compsilura* + Refuge from *Compsilura* models, respectively.

None of the four diurnal species with sufficient sample sizes showed any trend in detection probability (Fig 3). With all lists of nocturnal species considered, seven species showed declines, six showed no change, and four showed increases in detection probability (Fig 3). Removing lists from *Compsilura* refuge areas resulted in *Eumorpha pandorus* changing from a significant to a non-significant decline (the changed 95% confidence intervals overlapped zero), *Manduca sexta* (a crop pest) changing from a non-significant to a significant decline, and no changes in the presence, absence or direction of trends for the remaining 15 species (Fig 3). Thus eight species showed declines in at least one of the analyses.

Of the four species that were recorded too infrequently for statistical analysis, two or three appear to have declined. We found no records for *Manduca jasminearum* in Connecticut, its northeastern distributional limit where it was formerly regularly recorded, after 1963. This species persists farther west and south as it was reported in northern New Jersey in 2012, the last year of the study. There were no records of *Sphinx lucitiosa*, a northern species, in Connecticut later than 1957 or in Massachusetts later than 1973, whereas records for New Hampshire and Maine continue through 1999. Thus these two species appear to have declined sharply in parts of their ranges. *S*. *canadensis* is a northern species that was rarely recorded in the study area outside of Maine. *Proserpinus flavofasciata* is a small, diurnal, northern species for which we encountered only two records, both from Maine (in 1928 and 1991), in our study.

The climate variables included in the model contributed somewhat to explaining variance in hawk moth detection probability. For all species, all three climate variables were selected in at least one model of the best model set considered, and so were useful predictors of detection probability. However, climate variables were significantly different from zero for only six of the 21 species with sufficient samples, and the signs of the coefficients were inconsistent across species (Table 2).

**Table 2:** Model coefficients for climate variables with associated confidence intervals in brackets, based on the full set of records (Exposed to *Compsilura* + Refuge from *Compsilura* localities). Coefficients in bold were significantly different from 0.

Species	Minimum temperature of the coldest month	Mean temperature of the warmest quarter	Precipitation during the warmest quarter
*Amphion floridensis*	0.41 (-0.47, 1.29)	-0.09 (-1.1, 0.92)	-0.05 (-0.56, 0.46)
*Hemaris diffinis*	2.35 (-3.83, 8.52)	-2.19 (-8.29, 3.9)	-0.31 (-1.34, 0.71)
*Hemaris gracilis*	-3.76 (-8.97, 1.44)	-4.25 (-10.04, 1.54)	2 (-1.03, 5.02)
*Hemaris thysbe*	-0.38 (-1.96, 1.21)	0.71 (-0.67, 2.09)	0.36 (-0.25, 0.98)
*Darapsa choerilus*	**0.35 (0.01, 0.68)**	0.08 (-0.32, 0.47)	**-0.3 (-0.54, -0.06)**
*Darapsa myron*	0.24 (-0.13, 0.61)	0.13 (-0.25, 0.5)	0.18 (-0.06, 0.43)
*Darapsa versicolor*	0.02 (-0.66, 0.69)	0.51 (-0.07, 1.09)	0.07 (-0.32, 0.45)
*Deidamia inscriptum*	-0.07 (-0.43, 0.3)	0.09 (-0.28, 0.46)	-0.06 (-0.27, 0.15)
*Dolba hyloeus*	-0.27 (-0.58, 0.04)	0.22 (-0.07, 0.52)	0.13 (-0.08, 0.34)
*Eumorpha achemon*	0.31 (-0.41, 1.02)	0.31 (-0.31, 0.93)	0.31 (-0.17, 0.8)
*Eumorpha pandorus*	0.18 (-0.28, 0.65)	**0.43 (0.05, 0.8)**	-0.1 (-0.34, 0.14)
*Hyles gallii*	-0.31 (-1.1, 0.48)	**-1.37 (-2.19, -0.55)**	0.32 (-0.29, 0.92)
*Hyles lineata*	-0.25 (-1.03, 0.52)	0.43 (-0.26, 1.12)	0.16 (-0.32, 0.63)
*Lintneria eremitus*	0.49 (-0.32, 1.3)	0.83 (-0.02, 1.68)	0.08 (-0.33, 0.48)
*Manduca quinquemaculatus*	-0.08 (-0.71, 0.56)	0.36 (-0.1, 0.82)	0.11 (-0.3, 0.52)
*Manduca sexta*	0.32 (-0.1, 0.74)	0.3 (-0.07, 0.68)	0.04 (-0.24, 0.33)
*Sphecodina abbottii*	-0.36 (-0.78, 0.06)	-0.31 (-0.78, 0.15)	0.03 (-0.24, 0.3)
*Sphinx chersis*	**-0.51 (-0.92, -0.09)**	-0.05 (-0.53, 0.43)	0.24 (-0.12, 0.6)
*Sphinx drupiferarum*	0.52 (-0.12, 1.16)	**-0.87 (-1.47, -0.27)**	0.26 (-0.1, 0.62)
*Sphinx gordius/S*. *poecila*	0.2 (-0.19, 0.6)	-0.27 (-0.66, 0.11)	**-0.25 (-0.48, -0.01)**
*Sphinx kalmiae*	-0.43 (-0.9, 0.04)	-0.38 (-0.84, 0.09)	-0.19 (-0.46, 0.09)

**Fig 4:**
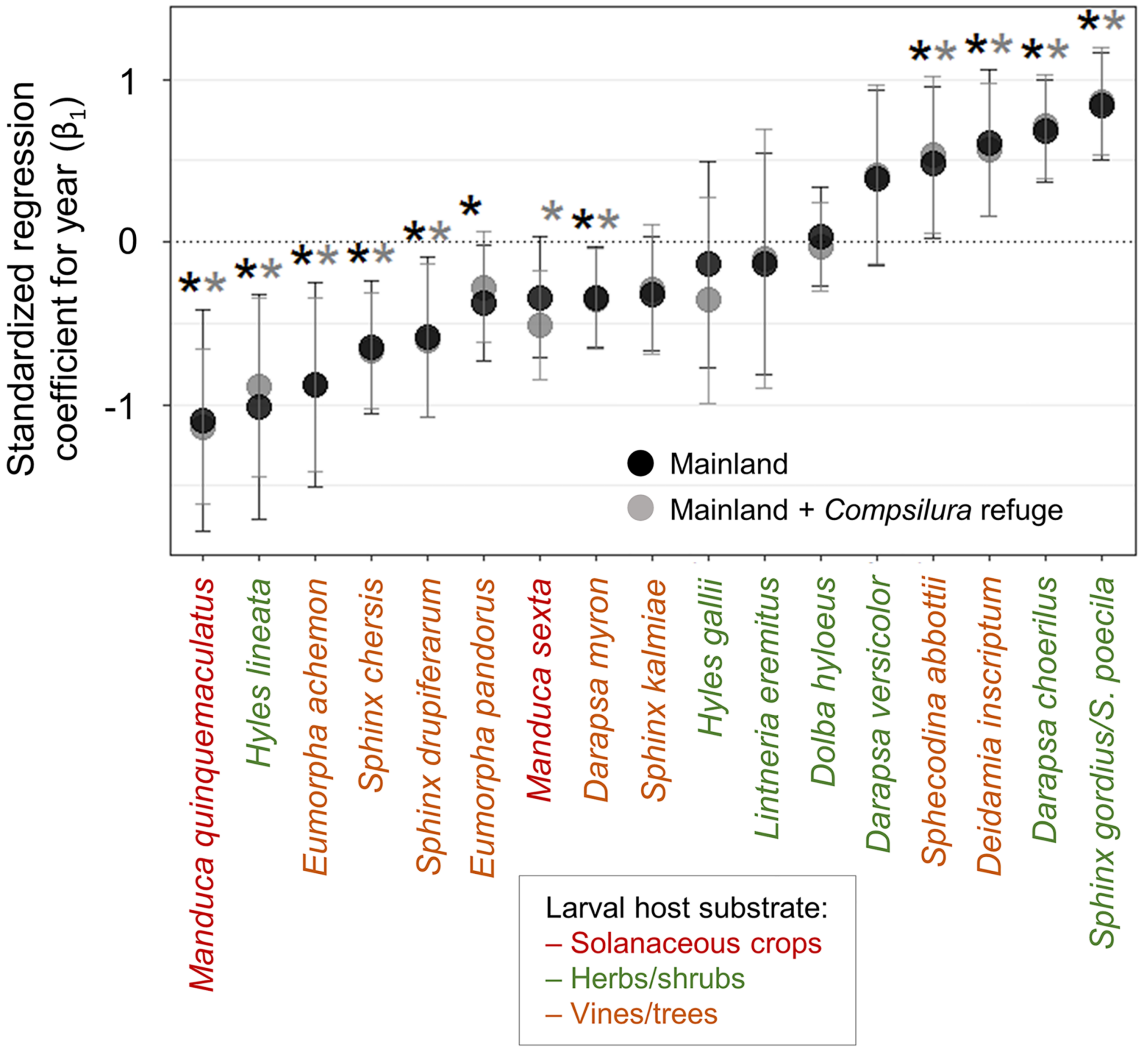
Temporal change in the area of tobacco and tomato planted in New England. Area charted in 5-yr intervals. Source: [59], [60]; data for tomatoes were not available for 1900, 2005, and 2010.

The area planted in tobacco or tomato on New England farms peaked in the 1920s, dropped precipitously during the Great Depression in the 1930s, rebounded until 1950, then declined again until leveling off after 1985 (Fig 4). The area planted in the two crops in the 2000s was 13% of the peak in the 1920s.

Among the nocturnal species for which there were sufficient records to analyze for trends (and ignoring the crop pests *Manduca sexta* and *M*. *quinquemaculatus* that feed in agricultural settings), five of the eight tree- or vine-feeding species declined whereas only one of the seven shrub- or herb-feeding species declined, a nonsignificant difference (two-tailed Fisher’s exact test, *P* = 0.12). Two of the tree/vine-feeding species that did not decline, *Deidamia inscriptum* and *Sphecodina abbottii*, are active only early in the season in May and June. Restricting the analysis to summer species (when *Compsilura* populations have built up) results in tree/vine feeders being more likely to decrease than shrub/herb feeders (two-tailed Fisher’s exact test, *P* = 0.029).

## Discussion

### Inferred population trends

Using existing records of hawk moths in museum and private collections over a 112-year period, we were able to detect statistical declines in eight species of northeastern U.S. hawk moth pollinators, or 38% of the species for which sample sizes were sufficient to allow analysis. Two additional species declined or disappeared altogether from portions of the study area, and a third, *Sphinx canadensis*, may have declined in the portion of its range that overlaps the study area. Thus, as many as 44% of the species examined appear to have declined in at least part of the Northeast. Just four species, 19% of species with sufficient sample sizes, increased over the study period.

Three patterns are evident from the results. First, all four diurnal hawk moth species showed no discernible trend in detection probability. Second, the two solanaceous crop pest species declined during the study period, consistent with the ten-fold decrease in tobacco and tomato farming in the region. Third, of the remaining 15 species with sufficient records for analysis, all but one of the six vine- or tree-feeding species that are active during the summer months declined. All but one of the nine species that are active only in the spring or feed on shrubs or herbs either showed no trend or increased during the study period. This latter result is consistent with the hypothesis that *Compsilura* parasitoidism has played a role in the declines. However, removing the numerous records from the *Compsilura* refuge did not lead to a consistent pattern of increased declines as would be expected if *Compsilura* were widely responsible for declines.

We note further that the species that declined tend to have large-bodied late instar larvae, with five of the six species having forewing lengths of at least 38 mmha, whereas the forewing length of all the species that increased is less than that length. We cannot say whether large size contributes directly to susceptibility to *Compsilura* attack or is related to another aspect of these species such as foraging substrate that confers susceptibility. The body size of the species in our sample appears to be associated with phylogeny [70], as large bodied species are restricted to just three genera, *Eumorpha*, *Manduca* and *Sphinx*. Without a larger sample of species, the effects of phylogeny and body size cannot be disentangled statistically [71].

These results are consistent with previous reports. Each of the eight species recorded as declining in this study were previously suspected to be declining in Connecticut [33]. Similarly, the four species that showed significant increases in this study were reported to be common in Connecticut [33]. *Sphinx chersis*, a species found to have declined in this study, was reported to be rare by [32]. [32] also reported no generalized widespread trend in *Hemaris gracilis* numbers, which agrees with our finding of no long-term trend in detection probability for this species.

Two of the species for which insufficient records were available for statistical analysis were previously reported as extirpated in Connecticut: *Manduca jasminearum* and *Sphinx lucitiosa* [33]. Our compilation is consistent with this observation, although the former species has recently been rediscovered in south-central Connecticut (D. Wagner, personal communication). *M*. *jasminearum* appears to have contracted from much of the extreme northern part of its range (Connecticut), although our records indicate that it persists in nearby northeastern New Jersey. *S*. *lucitiosa* appears to have disappeared from the southeastern edge of its range in southern New England and northeastern New Jersey. Considering that at least eight collectors have surveyed extensively for hawk moths in southern New England over the last several decades [33], the rarity of recent records suggests that the two species have declined in this region.

This study contributes to a growing body of evidence implicating the introductions of *Compsilura* in the early 1900s as a contributor to the declines of large moths in the northeastern U.S. The existence of a refuge from the parasitoid in Cape Cod and offshore islands [42], [31], [35] and the results of field experiments in which larvae of a suitable host species (the browntail moth, *Euproctis chrysorrhoea*) placed in historical habitats were heavily parasitized [38], [42] provide striking evidence in support of this contention. Our observations that declining species were those that feed as larvae in microhabitats or seasons when they are most exposed to *Compsilura* attack is an additional indication that introductions of the parasitoid have had broad effects on Northeastern lepidopteran abundance. Additional experimentation, such as tethering larvae from increasing and decreasing hawk moth species on different substrates (e.g., trees, vines, shrubs, herbs) and observing rates of *Compsilura* attack, would help confirm the patterns suggested by our results.

Both *Manduca sexta* and *M*. *quinquemaculatus* are well-known pests to crops in the family Solanaceae [25]. Tobacco was the most widespread solanaceous commercial crop in the northeastern U.S., and the dramatic decline in tobacco and tomato farming in the mid-20^th^ century could help explain declines in these two species. Other than these two crops, another solanaceous crop planted in the study area, potatoes, is planted extensively in northern Maine, but this area is outside of the normal range of either *Manduca* species [72], [25]. These species may use other solanaceous plants that grow in more natural habitat as host plants as they do elsewhere in their range [25], although to our knowledge this has not been documented for our study area. The concordance of declines in both the moths and solanaceous crop farming is therefore suggestive, but other factors could have contributed to the declines.

### Potential biases from collection data

Inferring population trends from specimen records requires several assumptions to hold true, as described above. The assumption that collecting effort is constant [44] was demonstrably not the case in our sample. Moth collectors have argued that the advent of mercury-vapor lights for attracting moths in the mid-1960s increased collecting efficiency [30]. Indeed, we found that hawk moth records for the study area show a marked and sustained increase after this time period. However, because our analysis focused on detection probability during collecting events relative to other species, an overall increase in hawk moth collection would not have skewed the results for any one species unless it is substantially more or less likely to be attracted to lights than other species. To our knowledge, only one species in our sample, *Lintneria eremitus*, has been suggested to avoid light traps [10]. Moreover, the predominant trend was of decline, which is counter to the expectation of increases if collecting methods were responsible for the trends detected.

A related assumption that collecting effort is constant across species [44], is hard to evaluate. Hawk moths are collected by netting at flowers, using lights or bait, or searching for larvae on host plants [25]. Collectors’ objectives vary, resulting in the use of different methods at different times and places [73]. The large samples we compiled may have been sufficient to even out these imbalances if there weren’t temporal trends in the use of specific collecting methods. Separating analyses for diurnal and nocturnal species also reduces the influence of collecting method on trend detection.

Bias by collectors toward preserving large, showier, or rarer species is another concern [74], [75], [73]. If this bias remains constant over time, then trend analyses will be unaffected. However, if common species were once shunned but are now collected at the rate in which they are encountered, they would appear to have increased whereas rare species would appear to have declined. Although this bias cannot be ruled out completely, we note that rare species would be unlikely to have sample sizes large enough for statistical analysis. Also, the complete disappearance of species from the collecting record, as observed here for parts of the range of *Manduca jasminearum* and *Sphinx lucitiosa*, is strongly suggestive, especially when backed up by numerous collectors with decades of experience [33].

### Alternative hypotheses

Alternative hypotheses could potentially explain the trends that we observed. Largescale gypsy moth spraying with DDT and carbaryl has often been suggested as a factor [29], [30] but neither has been widely used in the last 20 years. The use of *Bacillus thuringiensis* to control gypsy moths is unlikely to be a widespread cause of declines due to its limited use both spatially and temporally such that native species could rebound between years when it is applied [38], [32]. Furthermore, [76] showed that even under high laboratory doses sensitivity varies drastically among native moth species, although they did not test any hawk moths.

Climate change has led to changes in butterfly ranges in the northeastern U.S. [50] and could be expected to influence hawk moths in our study area. However, the climate variables we examined were at best weakly related to detection probability of the moths studied. Climate change would tend to cause the retraction from the southern edges of ranges and northward range extensions [77]. Although we did not specifically attempt to detect this effect, we note that of the two regional declines that we documented, *Manduca jasminearum* was a retraction from the northern edge of its range (opposite the climate prediction). In the future, the longer warm periods brought about by climate change may be expected to benefit a multivoltine parasitoid such as *Compsilura* [78].

Two other factors, light pollution and land use change, have likely played at least a small role in the patterns revealed in this study, although the relative importance of each is difficult to confirm. Artificial lights can disrupt nocturnal moth behavior [79], but documentation of population-level effects is mostly lacking [32], [12]. In our study, many of the declines are geographically restricted [33], whereas artificial lights shine at night in many regions across the study area [38]. For example, lights appear to shine just as brightly on Martha’s Vineyard and Nantucket as on the nearby mainland [80], yet many large-bodied moths that have declined in the mainland remain readily observable on the islands. One possible test would be to examine whether night light data collected by satellites [81] and summarized by county is related to spatial patterns of hawk moth detection probability. None of the four diurnal hawk moths in our study declined, a result that is consistent with the light pollution hypothesis, but the small number of diurnal species precludes this observation from providing strong support.

During the study period, forest cover initially increased as farms were abandoned and the use of fuelwood declined, then decreased somewhat due to suburbanization [36], [37]. The effects of this profound change in land use on hawk moths is difficult to assess. Most host plants are forest species and therefore the major trend of increased forest cover may be expected to benefit many hawk moths. However, the extent to which foliage from younger plants in earlier successional stands may be more suitable for larval development than foliage from older plants growing in mature forests is unknown for most species examined. However, declines in *Hyles lineata*, a species that feeds on early successional plants, could be related to the transition from agricultural lands to forest.

Forest composition has changed due to fire suppression and more recently because of browsing by superabundant deer (*Odocoileus virginianus*) [36], [82]. Data on forest composition are limited mostly to intensively studied research plots in restricted areas [37], and therefore inference about plant species availability for larval foraging across the study area is impossible. Nevertheless, the recent arrival of the introduced emerald ash borer (*Agrilus planipennis*), a specialist *Fraxinus* feeder, will likely cause a major reduction in the availability of host plants for Oleaceae-specialist species in the northeastern U.S. (*Manduca jasminearum*, *Sphinx canadensis*, *S*. *chersis*, and *S*. *kalmiae*) [83].

### Ecological consequences

Incomplete natural history information about hawk moth pollination challenges our ability to predict the ecological consequences of hawk moth declines in the northeastern U.S. Flower visitation by moths in general is poorly documented [12]. Efforts to document flower visitation by hawk moths is apparently limited to gardens or open disturbed areas, with few observations of flowers during nocturnal pollinator visits (e.g., [84], [85]). Plants reported as being visited by hawk moths are typically invasive weeds (e.g., *Lonicera japonica*, *Centaurea* spp.), cultivated plants (e.g., *Catharanthus roseus*, *Petunia* spp., *Phlox* spp., *Saponaria officionalis*) or native old field species (*Cirsium discolor*) [25], [85]. Clearly some hawk moths visit rare, native plants. For example, *Eumorpha achemon*, found to be declining in the study area, visits the threatened orchid *Platanthera praeclara* in the upper midwestern U.S. [10]. Whether native northeastern U.S. plants suffer pollen limitation as a result of hawk moth declines is unknown, but possible. The hawk moth species found in this study to be declining have noticeably longer tongue lengths (all but one species have tongues longer than 35 mm) than the species that increased (range 12–27 mm; no data for *Darapsa versicolor*) [86], [87], suggesting that the species that are becoming relatively more common may not be ecological replacements for the declining species. Beyond pollination, hawk moths and their larvae also serve as prey to nocturnal birds and other predators. Populations of these species, such as the eastern whip-poor-will (*Antrostomus vociferous*), which feeds on a variety of large-bodied crepuscular and nocturnal insects such as the species we examined [88] and is declining throughout the northeastern U.S. [89], may be negatively affected by losses in their prey base.

## Conclusions

This study demonstrates that concern over pollinator declines should be extended to hawk moths. At least ten of the 26 species examined, or more than one-third, were found to be in long-term decline or locally extirpated, whereas four species increased from 1900–2012 in the northeastern U.S. Although the causes for the increases are unknown, one factor in the declines appears to be mortality caused by the unintentional effects of the introduced biocontrol agent *Compsilura concinnata*. Changing agricultural practices may have played a role in the decline of two species recognized as crop pests. Other factors surely are at play, and careful monitoring and experimentation could determine their roles and importance. These results are cause for concern about the ecological integrity of the habitats where these moths once were plentiful as well as for the potential that specific native plant species may decline due to pollinator limitation. Moreover, the ecological consequences may extend to species that prey on moths.

## Supporting information

S1 Table Multicollinearity among model predictor variables assessed using variance inflation factors. (DOCX)

S2 Table Candidate models explaining the reporting rate of hawkmoths. Numbers correspond to numbers in S3 Table. Only models selected in the best predictor set of at least one species are shown. All models also included a spatial random effect of county.
(DOCX)

S3 Table Summary of model selection for 21 species of hawkmoths. Indicated are the number and, in brackets, the weight of evidence (AICw) corresponding to each model selected in the best model set. Model numbers correspond to models in S2 Table. Models shown were generated using the full set of records for each species.
(DOCX)

## References

[1] Biesmeijer JC, Roberts SP, Reemer M, Ohlemüller R, Edwards M, Peeters T, et al Parallel declines in pollinators and insect-pollinated plants in Britain and the Netherlands. Science. 2006;313:351–354. doi: 10.1126/science.1127863 16857940 10.1126/science.1127863

[2] Watanabe ME. Pollinators at risk. BioSci. 2013;64:5–10.

[3] IPBES. Summary for policymakers of the assessment report of the Intergovernmental Science-Policy Platform on Biodiversity and Ecosystem Services on pollinators, pollination and food production. Potts S.G. et al, editors. Secretariat of the Intergovernmental Science-Policy Platform on Biodiversity and Ecosystem Services, Bonn, Germany; 2016.

[4] Colla SR, Packer L. Evidence for decline in eastern North American bumblebees (Hymenoptera: Apidae), with special reference to Bombus affinis Cresson. Biodivers Conserv. 2008;17(6):1379–1391.

[5] Cameron SA, Lozier JD, Strange JP, Koch JB, Cordes N, Solter LF, et al Patterns of widespread decline in North American bumble bees. Proc Natl Acad Sci U S A. 2011 1 11;108(2):662–7. doi: 10.1073/pnas.1014743108 21199943 10.1073/pnas.1014743108 PMC3021065

[6] Bartomeus I, Ascher JS, Gibbs J, Danforth BN, Wagner DL, Hedtke SM, Winfree R. Historical changes in northeastern US bee pollinators related to shared ecological traits. Proc Natl Acad Sci U S A. 2013 3 19;110(12):4656–60. doi: 10.1073/pnas.1218503110 Epub 2013 Mar 4. 23487768 10.1073/pnas.1218503110 PMC3606985

[7] Federal Pollinator Health Task Force. National Strategy to Promote the Health of Honey Bees and Other Pollinators. 2015. https://www.epa.gov/pollinator-protection/federal-pollinator-health-task-force-epas-role.

[8] Atwater MM. Diversity and nectar hosts of flower-settling moths within a Florida sandhill ecosystem. J Nat Hist. 2013;47:2719–2734.

[9] Banza P, Belo ADF, Evans DM. The structure and robustness of nocturnal Lepidopteran pollen-transfer networks in a Biodiversity Hotspot. Insect Conserv Divers. 2015 doi: 10.1111/icad.12134

[10] Fox K, Vitt P, Anderson K, Fauske G, Travers S, Vik D, et al Pollination of a threatened orchid by an introduced hawk moth species in the tallgrass prairie of North America. Biol Conserv. 2013;167:316–324.

[11] Borkowsky C, Westwood AR. Seed capsule production in the endangered Western Prairie Fringed Orchid (*Platanthera praeclara*) in relation to sphinx moth (Lepidoptera: Sphingidae) activity. J Lepid Soc. 2009;63:110–117.

[12] Hahn M, Brühl CA. The secret pollinators: an overview of moth pollination with a focus on Europe and North America. Arthropod Plant Interact. 2016;10:21–28.

[13] Fox K, Anderson KM, Andres R, Foster MC, Foster CE, Vik D, et al Nectar Robbery and Thievery in the Hawk Moth (Lepidoptera: Sphingidae)-Pollinated Western Prairie Fringed Orchid Platanthera praeclara. Ann Entomol Soc Am. 2015 10 1;108(6):1000–13.

[14] MacGregor CJ, Pocock MJ, Fox R, Evans DM. Pollination by nocturnal Lepidoptera, and the effects of light pollution: a review. Ecol Entomol. 2015 6;40(3):187–198. doi: 10.1111/een.12174 25914438 10.1111/een.12174 PMC4405039

[15] Faegri K, van der Pijl L. The principles of pollination ecology. 3rd Rev ed. Oxford: Pergamon Press; 1979.

[16] Gritsky G. Darwin's Madagascan hawk moth prediction. Am Entomol. 1991;37:206–210.

[17] Johnson SD, Moré M, Amorim FW, Haber WA, Frankie GW, Stanley DA, et al The long and the short of it: a global analysis of hawkmoth pollination niches and interaction networks. Funct Ecol. 2016 doi: 10.1111/1365-2435.12753 28344378 10.1111/1365-2435.12753 PMC5363726

[18] Grunert LW, Clarke JW, Ahuja C, Eswaran H, Nijhout HF. A quantitative analysis of growth and size regulation in Manduca sexta: the physiological basis of variation in size and age at metamorphosis. PloS one. 2015 5 26;10(5):e0127988 doi: 10.1371/journal.pone.0127988 26011714 10.1371/journal.pone.0127988 PMC4444085

[19] Hayes MB, Jiao L, Tsao TH, King I, Jennings M, Hou C. High temperature slows down growth in tobacco hornworms (Manduca sexta larvae) under food restriction. Insect Sci. 2015 3 1;22(3):424–30. doi: 10.1111/1744-7917.12109 24459098 10.1111/1744-7917.12109

[20] Schreiber H. Dispersal centres of Sphingidae (Lepidooptera) in the Neotropical region. Biogeographica 10. The Hague-Boston; 1978.

[21] Haber WA, Frankie GW. A tropical hawkmoth community: Costa Rican dry forest Sphingidae. Biotropica. 1989;21:155–172.

[22] Nilsson LA. The evolution of flowers with deep corolla tubes. Nature. 1988 7 14;334(6178):147–9.

[23] Whittall JB, Hodges SA. Pollinator shifts drive increasingly long nectar spurs in columbine flowers. Nature. 2007 6 7;447(7145):706 doi: 10.1038/nature05857 17554306 10.1038/nature05857

[24] Ollerton J, Alarcón R, Waser NM, Price MV, Watts S, Cranmer L, et al A global test of the pollination syndrome hypothesis. Ann Bot. 2009 6;103(9):1471–80. doi: 10.1093/aob/mcp031 19218577 10.1093/aob/mcp031 PMC2701765

[25] Tuttle JP. The hawk moths of North America. Washington, DC: Wedge Entomological Foundation; 2007.

[26] Grant V. The systematic and geographical distribution of hawkmoth flowers in the temperate North American flora. Bot Gaz. 1983;144:439–449.

[27] Sheviak CJ, Bowles ML. The prairie fringed orchids: a pollinator-isolated species pair. Rhodora. 1986;88:267–290.

[28] Nilsson LA. The evolution of flowers with deep corolla tubes. Nature. 1988;334:147–149.

[29] Hessel SA. A preliminary scan of rare and endangered Nearctic moths. Atala. 1976;4:19–21.

[30] Schweitzer DF. Status of Saturniidae in the northeastern USA: A quick review. News Lepid Soc. 1988;1(January- February):4–5.

[31] Goldstein PZ. Life history of the imperial moth *Eacles imperialis* (Drury) (Saturniidae: Ceratocampinae) in New England, U.S.A.: distribution, decline, and nutritional ecology of a relictual islandic population. J Lepid Soc. 2010;42:34–49.

[32] Schweitzer DF, Minno MC, Wagner DL. Rare, declining, and poorly known butterflies and moths (Lepidoptera) of forests and woodlands in the Eastern United States. Morgantown (WV): U.S. Forest Service, Forest Health Technology Enterprise Team; 2011. FHTET-2011-01.

[33] Wagner DL. Moth decline in the northeastern United States. News Lepid Soc. 2012;54:52–56.

[34] St. Laurent R. Updated distributional data for *Citheronia sepulcralis* Grote & Robinson, 1865 (Saturniidae: Ceratocampinae), with a new host plant record. J Lepid Soc. 2016;70:9–14.

[35] Goldstein PZ, Morita S, Capshaw G. Stasis and flux among Saturniidae and Sphingidae (Lepidoptera) on Massachusetts' offshore islands and the possible role of *Compsilura concinnata* (Meigen) (Diptera: Tachinidae) as an agent of mainland New England moth declines. Proc Entomol Soc Wash. 2015;117(3):347–366.

[36] Foster DR. Land-use history and four hundred years of vegetation change in New England Principles, patterns and processes of land use change: some legacies of the Columbian encounter. Scope Publication. Wiley, New York 1995:253–319.

[37] Foster DR, Aber JD, editors. Forests in time: the environmental consequences of 1,000 years of change in New England. Yale University Press; 2006 4 1.

[38] Boettner GH, Elkinton JS, Boettner CJ. Effects of a biological control introduction on three nontarget native species of saturniid moths. Conserv Biol. 2000;14:1798–1806. 10.1111/j.1523-1739.2000.99193.x 35701905

[39] Kellogg SK, Fink LS, Brower LP. Parasitism of native luna moths, Actias luna (L.)(Lepidoptera: Saturniidae) by the introduced Compsilura concinnata (Meigen)(Diptera: Tachinidae) in central Virginia, and their hyperparasitism by trigonalid wasps (Hymenoptera: Trigonalidae). Environ Entomol. 2003 10;32(5):1019–27.

[40] Arnaud PH Jr. A host-parasite catalog of North American Tachinidae (Diptera). U.S. Dep. Agric. Misc. Publ. 1978;1319:1–1860.

[41] Weseloh RM. Implications of tree microhabitat preferences of *Compsilura concinnata* (Diptera: Tachinidae) for its effectiveness as a gypsy moth parasitoid. Can Entomol. 1982;114(7):617–622.

[42] Elkinton JS, Parry D, Boettner GH. Implicating an introduced generalist parasitoid in the invasive browntail moth's enigmatic demise. Ecology. 2006 10;87(10):2664–72. 17089674 10.1890/0012-9658(2006)87[2664:iaigpi]2.0.co;2

[43] Nichols JD, Williams BK. Monitoring for conservation. Trends Ecol Evol. 2006 12 31;21(12):668–73. doi: 10.1016/j.tree.2006.08.007 16919361 10.1016/j.tree.2006.08.007

[44] McCarthy MA. Identifying declining and threatened species with museum data. Biol Conserv. 1998;83:9–17.

[45] Colla SR, Gadallah F, Richardson L, Wagner D, Gall L. Assessing declines of North American bumble bees (*Bombus* spp.) using museum specimens. Biodivers Conserv. 2012;21:3585–3595.

[46] Graham CH, Ferrier S, Huettman F, Moritz C, Peterson AT. New developments in museum-based informatics and applications in biodiversity analysis. Trends Ecol Evol. 2004 9 30;19(9):497–503. doi: 10.1016/j.tree.2004.07.006 16701313 10.1016/j.tree.2004.07.006

[47] Hortal J, Borges PA, Gaspar C. Evaluating the performance of species richness estimators: sensitivity to sample grain size. J Anim Ecol. 2006 1 1;75(1):274–87. 16903065

[48] Isaac NJ, Strien AJ, August TA, Zeeuw MP, Roy DB. Statistics for citizen science: extracting signals of change from noisy ecological data. Methods Ecol Evol. 2014 10 1;5(10):1052–60.

[49] Szabo JK, Vesk PA, Baxter PW, Possingham HP. Regional avian species declines estimated from volunteer-collected long-term data using List Length Analysis. Ecol Appl. 2010 12 1;20(8):2157–69. 21265449 10.1890/09-0877.1

[50] Breed GA, Stichter S, Crone EE. Climate-driven changes in northeastern US butterfly communities. Nat Clim Chang. 2013 2 1;3(2):142.

[51] Barnes M, Szabo JK, Morris WK, Possingham H. Evaluating protected area effectiveness using bird lists in the Australian Wet Tropics. Divers Distrib. 2015 4 1;21(4):368–78.

[52] Zeilinger AR, Rapacciuolo G, Turek D, Oboyski PT, Almeida RP, Roderick GK. Museum specimen data reveal emergence of a plant disease may be linked to increases in the insect vector population. Ecol Appl. 2017 4 29. 10.1002/eap.1569 28459124

[53] Rapacciuolo G, Ball-Damerow JE, Zeilinger AR, Resh VH. Detecting long-term occupancy changes in Californian odonates from natural history and citizen science records. Biodivers Conserv. 2017:1–7.

[54] Roberts RL, Donald PF, Green RE. Using simple species lists to monitor trends in animal populations: new methods and a comparison with independent data. Animal Conservation. 2007 8 1;10(3):332–9.

[55] Isaac NJ, Pocock MJ. Bias and information in biological records. Biol J Linn Soc. 2015 6 15;115(3):522–31.

[56] O’Donnell MS, Ignizio DA. Bioclimatic predictors for supporting ecological applications in the conterminous United States: U.S. Geological Survey Data Series 691;2012.

[57] Daly C, Halbleib M, Smith JI, Gibson WP, Doggett MK, Taylor GH, et al Physiographically sensitive mapping of climatological temperature and precipitation across the conterminous United States. Int J Climatol. 2008;28:2031–2064.

[58] PRISM Climate Group. PRISM gridded climate data. Oregon State University, Corvallis, OR 2013 http://prism.oregonstate.edu.

[59] United States Department of Agriculture (USDA) [Internet]. USDA National Agricultural Statistics Service (NASS): USDA Economics, Statistics and Market Information System c1975–2016. 2016. http://usda.mannlib.cornell.edu/MannUsda/viewDocumentInfo.do?documentID=1000.

[60] USDA [Internet]. Albert R. Mann Library, Cornell University: USDA Census of Agriculture Historical Archive c1840–2002. 2016. http://agcensus.mannlib.cornell.edu/AgCensus/homepage.do.

[61] Szabo JK, Vesk PA, Baxter PW, Possingham HP. Regional avian species declines estimated from volunteer-collected long-term data using List Length Analysis. Ecol Appl. 2010 12;20(8):2157–69. 21265449 10.1890/09-0877.1

[62] van Strien AJ, Termaat T, Kalkman V, Prins M, De Knijf G, Gourmand AL, et al Occupancy modelling as a new approach to assess supranational trends using opportunistic data: a pilot study for the damselfly *Calopteryx splendens*. Biodivers Conserv. 2013;22:673–686.

[63] Isaac NJB, van Strien AJ, August TA, de Zeeuw MP, Roy DB. Statistics for citizen science: extracting signals of change from noisy ecological data. Methods Ecol Evol. 2014;5:1052–1060.

[64] Schielzeth H. Simple means to improve the interpretability of regression coefficients. Methods Ecol Evol. 2010;1:103–113.

[65] Burnham KP, Anderson DR. Model selection and inference: a practical theoretic approach. New York: Springer-Verlag; 2002.

[66] Johnson JB, Omland KS. Model selection in ecology and evolution. Trends Ecol Evol. 2004 2;19(2):101–8. doi: 10.1016/j.tree.2003.10.013 16701236 10.1016/j.tree.2003.10.013

[67] Coyle JR, Hurlbert AH. Environmental optimality, not heterogeneity, drives regional and local species richness in lichen epiphytes. Glob Ecol Biogeogr. 2016;25:406–417.

[68] Bartoń K. MuMIn: multi-model inference, R package version 1.15.5; 2015.

[69] R Core Team. R: a language and environment for statistical computing. R Foundation for Statistical Computing, Vienna; 2016.

[70] Kawahara AY, Mignault AA, Regier JC, Kitching IJ, Mitter C (2009) Phylogeny and Biogeography of Hawkmoths (Lepidoptera: Sphingidae): Evidence from Five Nuclear Genes. PLoS ONE 4(5): e5719 doi: 10.1371/journal.pone.0005719 19492095 10.1371/journal.pone.0005719 PMC2683934

[71] Ives AR, Zhu J. Statistics for correlated data: phylogenies, space, and time. Ecol Appl. 2006 2;16(1):20–32. 16705958 10.1890/04-0702

[72] Schweitzer DF. Survival of freezing and subsequent summer eclosion by three migratory moths: *Manduca sexta* and *Hyles lineata* (Sphingidae), and *Helicoverpa zea* (Noctuidae). J Lepid Soc. 2006;60(2):101–103.

[73] Wehi PM, Whaanga H, Trewick SA. Artefacts, biology and bias in museum collection research. Mol Ecol. 2012 7;21(13):3103–9. 22916347 10.1111/j.1365-294x.2012.05589.x

[74] Klots AB. Notes on melanism on some Connecticut moths. J N Y Entomol Soc. 1964;72:142–144.

[75] Petersen FT, Meier R, Larsen MN. Testing species richness estimation methods using museum label data on the Danish Asilidae. Biodivers Conserv. 2003;12:687–701.

[76] Peacock JW, Schweitzer DF, Carter JL, Dubois NR. Laboratory assessment of the effects of *Bacillus thuringiensis* on native Lepidoptera. Environ Entomol. 1998;27:450–457.

[77] Parmesan C. Ecological and evolutionary responses to recent climate change. Annu Rev Ecol Evol Syst. 2006;37,637–669. doi: 10.1146/annurev.ecolsys.37.091305.110100

[78] Karuppaiah V, Sujayanad GK. Impact of climate change on population dynamics of insect pests. World Journal of Agricultural Sciences. 2012;8(3):240–6.

[79] Macgregor CJ, Evans DM, Fox R, Pocock MJO. The dark side of street lighting: impacts on moths and evidence for the disruption of nocturnal pollen transport. Glob Chang Biol. 2016 doi: 10.1111/gcb.13371 27251575 10.1111/gcb.13371

[80] National Aeronautics and Space Administration (NASA). NASA Visible Earth: Night Lights—Flat map. 2012. https://visibleearth.nasa.gov/view.php?id=79765.

[81] National Oceanic and Atmospheric Administration (NOAA). Defense Meteorological Satellite Program (DMSP). 2016. http://ngdc.noaa.gov/eog/dmsp.html.

[82] Schweitzer DJ, Garris R, McBride AE, Smith JAM. The current status of forest Macrolepidoptera in northern New Jersey: evidence for the decline of understory specialists. J Insect Conserv 2014 doi: 10.1007/s10841-014-9658-0

[83] Wagner DL, Todd KJ. Ecological impacts of emerald ash borer In: Van Driesche R, Reardon RC, editors. Biology and control of emerald ash borer. Morgantown (WV): USDA Forest Service; 2015 USDA Technical Bulletin FHTET-2014-09. p. 15–63.

[84] Lovell JH. The visitors of the Caprifoliaceae. Am Nat. 1900;34:37–51.

[85] Tartaglia ES, Handel SN. Nectar plant preferences of *Hemaris* (Sphingidae: Lepidoptera) on co-occurring native *Cirsium* and non-native *Centaurea* (Asteraceae) inflorescences. J Pollinat Ecol. 2014;13:184–187.

[86] Cuthrell, DL. Insects associated with the prairie fringe orchids, Platanthera praeclara Sheviak & Bowles and P. leucophaea (Nuttall) Lindley. Unpubl masters thesis 1997, North Dakota State Univ, Fargo, ND.

[87] Miller WE. Diversity and evolution of tongue length in hawkmoths(Sphingidae). J Lepid Soc. 1997;51(1):9–31.

[88] Bent AC. Life Histories of North American [birds]: Cuckoos, goatsuckers, hummingbirds and their allies. US Government Printing Office; 1940.

[89] Sauer JR, Hines JE, Fallon JE, Pardieck KJ, Ziolkowski DJ Jr, Link WA. The North American Breeding Bird Survey, Results and Analysis 1966–2013. Version 01.30.2015. USGS Patuxent Wildlife Research Center, Laurel, MD. 2014. https://www.mbr-pwrc.usgs.gov/bbs/bbs.html.

