# Supplementary material for: Are pollinating hawk moths declining in the Northeastern United States? An analysis of collection records
Source: PLoS One. 2017 Oct 5;12(10):e0185683. doi: 10.1371/journal.pone.0185683 (PMC5628844; doi:10.1371/journal.pone.0185683)
Supplement: S1 Table — (DOCX) [file pone.0185683.s001.docx]

| **Model variable** | **Variance Inflation Factor** |
| --- | --- |
| Year (*year*) | 1.31 |
| List length (*L*) | 1.27 |
| Number of records (*records*) | 1.21 |
| Bio6 | 2.09 |
| Bio10 | 1.68 |
| Bio18 | 1.04 |
