# Supplementary material for: Are pollinating hawk moths declining in the Northeastern United States? An analysis of collection records
Source: PLoS One. 2017 Oct 5;12(10):e0185683. doi: 10.1371/journal.pone.0185683 (PMC5628844; doi:10.1371/journal.pone.0185683)
Supplement: S2 Table — Numbers correspond to numbers in S3 Table. Only models selected in the best predictor set of at least one species are shown. All models also included a spatial random effect of county. (DOCX) [file pone.0185683.s002.docx]

| **Model** | **Model number** |
| --- | --- |
| records+L | 1 |
| bio10+records+L | 2 |
| bio18+records+L | 3 |
| bio10+bio18+records+L | 4 |
| bio6+records+L | 5 |
| bio10+bio6+records+L | 6 |
| bio18+bio6+records+L | 7 |
| bio10+bio18+bio6+records+L | 8 |
| year+records+L | 9 |
| bio10+year+records+L | 10 |
| bio18+year+records+L | 11 |
| bio10+bio18+year+records+L | 12 |
| bio6+year+records+L | 13 |
| bio10+bio6+year+records+L | 14 |
| bio18+bio6+year+records+L | 15 |
| bio10+bio18+bio6+year+records+L | 16 |
