# Supplementary material for: Are pollinating hawk moths declining in the Northeastern United States? An analysis of collection records
Source: PLoS One. 2017 Oct 5;12(10):e0185683. doi: 10.1371/journal.pone.0185683 (PMC5628844; doi:10.1371/journal.pone.0185683)
Supplement: S3 Table — Indicated are the number and, in brackets, the weight of evidence (AICw) corresponding to each model selected in the best model set. Model numbers correspond to models in S2 Table. Models shown were generated using the full set of records for each species. (DOCX) [file pone.0185683.s003.docx]

| **Species** | **Models selected** |
| --- | --- |
| *Amphion floridensis* | 1 (0.26), 5 (0.15), 2 (0.1), 3 (0.09), 9 (0.08), 6 (0.07), 7 (0.05), 13 (0.05), 4 (0.03), 10 (0.03), 11 (0.03), 8 (0.02), 14 (0.02), 15 (0.01), 12 (0.26), 16 (0.15) |
| *Darapsa choerilus* | 15 (0.47), 16 (0.17), 11 (0.14), 12 (0.11), 13 (0.07), 14 (0.03), 10 (0.47), 9 (0.17), 7 (0.14), 5 (0.11), 8 (0.07), 6 (0.03), 4 (0.47), 2 (0.17), 3 (0.14), 1 (0.11) |
| *Darapsa myron* | 15 (0.15), 11 (0.14), 9 (0.14), 13 (0.13), 12 (0.09), 10 (0.07), 16 (0.06), 14 (0.05), 1 (0.05), 3 (0.03), 5 (0.02), 2 (0.02), 7 (0.02), 4 (0.02), 6 (0.01), 8 (0.15) |
| *Darapsa versicolor* | 10 (0.17), 2 (0.15), 9 (0.12), 1 (0.07), 14 (0.07), 12 (0.07), 4 (0.06), 6 (0.05), 13 (0.05), 11 (0.04), 5 (0.04), 3 (0.03), 16 (0.03), 8 (0.02), 15 (0.02), 7 (0.02) |
| *Deidamia inscriptum* | 9 (0.35), 11 (0.15), 10 (0.14), 13 (0.13), 12 (0.06), 14 (0.06), 15 (0.06), 16 (0.02), 1 (0.35), 2 (0.15), 5 (0.14), 3 (0.13), 6 (0.06), 4 (0.06), 7 (0.06), 8 (0.02) |
| *Dolba hyloeus* | 6 (0.17), 8 (0.12), 1 (0.11), 3 (0.08), 5 (0.07), 2 (0.06), 14 (0.06), 4 (0.05), 7 (0.05), 9 (0.04), 16 (0.04), 11 (0.03), 13 (0.03), 10 (0.03), 12 (0.02), 15 (0.02) |
| *Eumorpha achemon* | 9 (0.21), 11 (0.17), 10 (0.15), 13 (0.13), 12 (0.11), 15 (0.11), 14 (0.06), 16 (0.05), 1 (0.21), 3 (0.17), 2 (0.15), 5 (0.13), 4 (0.11), 7 (0.11), 6 (0.06), 8 (0.05) |
| *Eumorpha pandorus* | 10 (0.22), 2 (0.17), 12 (0.11), 14 (0.1), 4 (0.09), 6 (0.06), 16 (0.05), 13 (0.05), 8 (0.03), 15 (0.02), 5 (0.02), 9 (0.02), 1 (0.02), 3 (0.01), 11 (0.01), 7 (0.01) |
| *Hemaris diffinis* | 1 (0.25), 5 (0.11), 3 (0.1), 6 (0.09), 9 (0.08), 2 (0.08), 7 (0.04), 14 (0.04), 13 (0.04), 11 (0.04), 4 (0.03), 8 (0.03), 10 (0.02), 15 (0.01), 16 (0.25), 12 (0.11) |
| *Hemaris gracilis* | 8 (0.16), 7 (0.16), 4 (0.14), 6 (0.14), 2 (0.1), 5 (0.07), 15 (0.05), 12 (0.04), 14 (0.04), 16 (0.04), 10 (0.03), 13 (0.02), 1 (0.16), 9 (0.16), 3 (0.14), 11 (0.14) |
| *Hemaris thysbe* | 1 (0.19), 3 (0.13), 2 (0.12), 6 (0.08), 4 (0.07), 5 (0.07), 9 (0.06), 7 (0.05), 11 (0.04), 10 (0.04), 8 (0.04), 12 (0.03), 14 (0.03), 13 (0.02), 15 (0.01), 16 (0.01) |
| *Hyles gallii* | 2 (0.25), 10 (0.16), 4 (0.14), 6 (0.13), 12 (0.13), 14 (0.07), 8 (0.07), 16 (0.05), 5 (0.25), 13 (0.16), 7 (0.14), 15 (0.13), 11 (0.13), 9 (0.07), 1 (0.07), 3 (0.05) |
| *Hyles lineata* | 9 (0.27), 10 (0.19), 11 (0.12), 14 (0.12), 13 (0.1), 12 (0.09), 16 (0.05), 15 (0.05), 1 (0.27), 5 (0.19), 6 (0.12), 2 (0.12), 3 (0.1), 7 (0.09), 8 (0.05), 4 (0.05) |
| *Lintneria eremitus* | 2 (0.25), 6 (0.16), 4 (0.1), 5 (0.08), 10 (0.08), 8 (0.06), 14 (0.06), 1 (0.05), 7 (0.03), 12 (0.03), 13 (0.03), 16 (0.02), 3 (0.02), 9 (0.02), 15 (0.25), 11 (0.16) |
| *Manduca quinquemaculatus* | 10 (0.27), 9 (0.23), 14 (0.12), 12 (0.11), 11 (0.1), 13 (0.09), 16 (0.05), 15 (0.04), 1 (0.27), 5 (0.23), 2 (0.12), 3 (0.11), 6 (0.1), 7 (0.09), 4 (0.05), 8 (0.04) |
| *Manduca sexta* | 10 (0.22), 13 (0.2), 14 (0.17), 9 (0.08), 12 (0.08), 15 (0.08), 16 (0.06), 11 (0.03), 2 (0.02), 1 (0.01), 5 (0.22), 6 (0.2), 4 (0.17), 3 (0.08), 8 (0.08), 7 (0.08) |
| *Sphecodina abbottii* | 13 (0.22), 10 (0.16), 14 (0.14), 9 (0.09), 15 (0.08), 12 (0.06), 16 (0.05), 5 (0.04), 1 (0.04), 11 (0.03), 2 (0.03), 6 (0.02), 7 (0.02), 3 (0.01), 4 (0.22), 8 (0.16) |
| *Sphinx chersis* | 13 (0.34), 15 (0.27), 14 (0.13), 16 (0.1), 10 (0.05), 9 (0.05), 12 (0.03), 11 (0.03), 5 (0.34), 7 (0.27), 6 (0.13), 2 (0.1), 1 (0.05), 8 (0.05), 4 (0.03), 3 (0.03) |
| *Sphinx drupiferarum* | 16 (0.28), 14 (0.24), 10 (0.19), 12 (0.17), 2 (0.04), 6 (0.03), 4 (0.02), 8 (0.01), 11 (0.28), 9 (0.24), 15 (0.19), 13 (0.17), 3 (0.04), 1 (0.03), 7 (0.02), 5 (0.01) |
| *Sphinx gordius* | 11 (0.26), 12 (0.2), 16 (0.18), 15 (0.11), 9 (0.09), 10 (0.06), 14 (0.05), 13 (0.04), 5 (0.26), 7 (0.2), 6 (0.18), 8 (0.11), 1 (0.09), 3 (0.06), 2 (0.05), 4 (0.04) |
| *Sphinx kalmiae* | 5 (0.09), 13 (0.09), 7 (0.08), 10 (0.07), 4 (0.07), 2 (0.07), 8 (0.07), 15 (0.07), 14 (0.06), 12 (0.06), 16 (0.06), 6 (0.06), 1 (0.05), 9 (0.04), 3 (0.03), 11 (0.02) |
